# Multiple cardiac calcified amorphous tumors with morphologically different characteristics complicated by aortic regurgitation: a case report

**DOI:** 10.1186/s44215-022-00028-9

**Published:** 2023-03-28

**Authors:** Hironobu Sakurai, Takeshi Someya, Satoshi Yamamoto, Eisaku Ito, Hidehito Kuroki, Toshizumi Shirai

**Affiliations:** 1 Department of Cardiothoracic Surgery, Ome Municipal General Hospital, 4-16-5, Higashiome, Ome City, Tokyo 198-0042 Japan; 2 Department of Pathology, Ome Municipal General Hospital, 4-16-5, Higashiome, Ome City, Tokyo Japan

**Keywords:** Calcified amorphous tumor, Cardiac tumor, Pathology, Aortic regurgitation, Mitral annular calcification

## Abstract

**Background:**

Cardiac calcified amorphous tumors (CATs) are non-neoplastic cardiac tumors of unknown origin and etiology. Several simultaneous CATs rarely occur in multiple cardiac chambers. Although CATs carry a benign prognosis, they have a risk of complications such as systemic embolism.

**Case presentation:**

We report the case of a 79-year-old woman with two CATs and aortic regurgitation due to perforations of aortic cusps. She underwent surgical tumor resection with aortic valve replacement. The CATs were macroscopically and histologically different, which may suggest different developmental stages. One CAT was in the left atrium; it was less mobile and had nodular calcifications within dense fibrous tissue. The other CAT was in the left ventricular outflow tract; it was highly mobile, with nodular calcifications surrounded by amorphous fibrin and sanguineous deposits. The highly mobile CAT mechanically damaged the aortic cusps and caused perforations. The patient has survived over 2 years with no recurrence of the cardiac masses on echocardiography.

**Conclusion:**

The patient underwent surgical resection for two CATs. The tumors occurred in different areas and had different macroscopic and histological characteristics. We recommend early resection for highly mobile CATs because of the high risks of embolization and injuries to the surrounding tissues.

## Background

Calcified amorphous tumors (CATs) are rare, non-neoplastic cardiac tumors that can occur in any cardiac chamber [1–3]. Multiple simultaneous CATs have been described previously; however, such cases are infrequent [4–6]. Histological examinations of CATs have revealed calcified nodules surrounded by amorphous materials [1]. Herein, we describe the case of a patient with multiple CATs treated with surgical resection. The CATs had different macroscopic and histological characteristics. One CAT was highly mobile and caused aortic regurgitation due to aortic cusp perforations.

## Case presentation

**Fig. 1:**
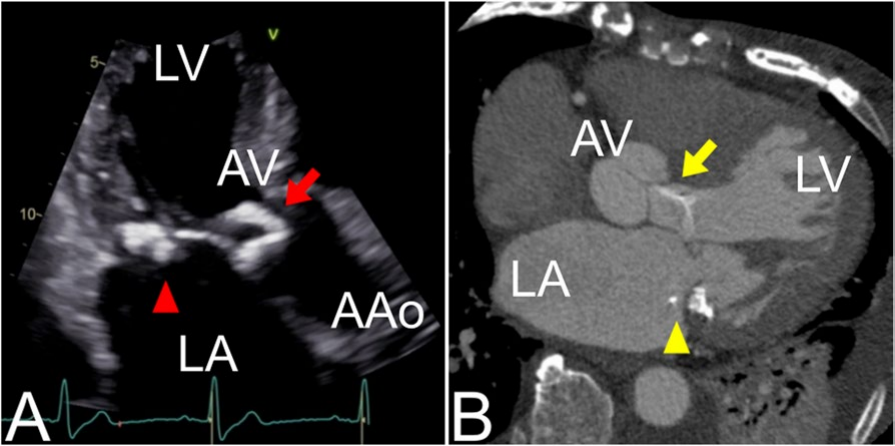
Preoperative transthoracic echocardiography and computed tomography images. **A** Transthoracic echocardiography reveals a highly echoic mass on the mitral annulus (red arrowhead) and a highly echoic and mobile mass in the left ventricle outflow tract (red arrow). **B** Computed tomography reveals a calcified mass in the mitral annulus (yellow arrowhead) and a linear calcification in the left ventricle (yellow arrow). AAo, ascending aorta; AV, aortic valve; LA, left atrium; LV, left ventricle

A 79-year-old woman with a history of hypertension and nephrosclerosis, which required hemodialysis for 8 years, presented with dyspnea caused by new-onset heart failure. Informed consent was obtained via a formal written waiver. Echocardiography revealed moderate aortic regurgitation and two highly echoic masses; one in the left atrium (LA) and the other in the left ventricular outflow tract (LVOT) (Fig. 1A).

Echocardiography conducted the previous year had not shown any cardiac mass or aortic regurgitation. The LA mass arose from the mitral annular calcification (MAC) of the posterior mitral leaflet. The LVOT mass was string-like, swung widely, and protruded through the aortic valve into the ascending aorta during systole. Computed tomography revealed a calcified mass in the mitral annulus with heavy MAC and linear calcification in the LVOT that extended to the aortic valve (Fig. 1B). Brain magnetic resonance imaging revealed multiple cerebral infarctions. Surgical resection of the cardiac masses was performed to prevent further embolization and determine an accurate diagnosis.

**Fig. 2:**
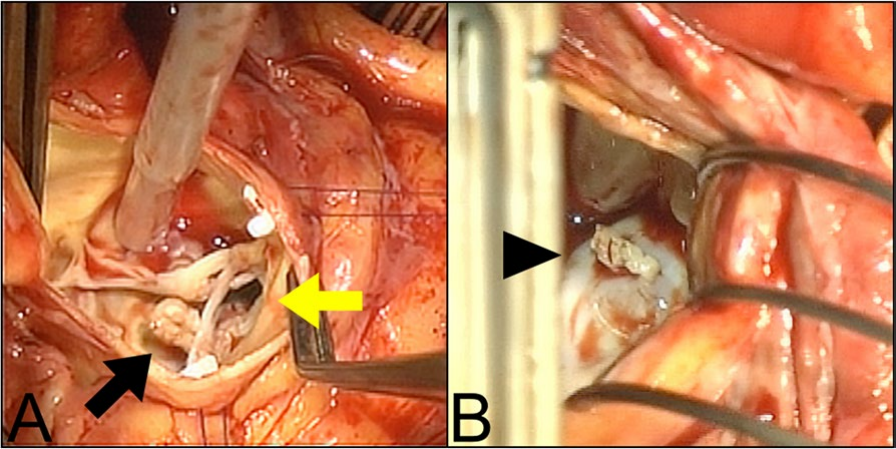
Intraoperative photographs. The masses in the left ventricle outflow tract **A**, black arrow and the left atrium **B**, black arrowhead; large perforations of non- and right-cusps **A**, yellow arrow causing aortic regurgitation

After median sternotomy, cardiopulmonary bypass was established using arterial cannulation of the ascending aorta and venous cannulations of the superior and inferior vena cava. The aorta was cross-clamped, followed by a routine aortic incision. The LVOT mass was connected to the chordae tendineae of the mitral valve and extended into the aortic valve (Fig. 2A). This was completely resected from the chordae tendineae without causing damage. The aortic valve was tricuspidate with calcifications and had large perforations of the right- and non-coronary cusps, causing aortic regurgitation. Therefore, aortic valve replacement was performed with a bioprosthetic valve. The mitral valve was inspected through a right-sided left atrial incision. The LA mass, adhered to the posterior MAC (Fig. 2B), was completely removed from the annulus, preserving the valvular function.

**Fig. 3:**
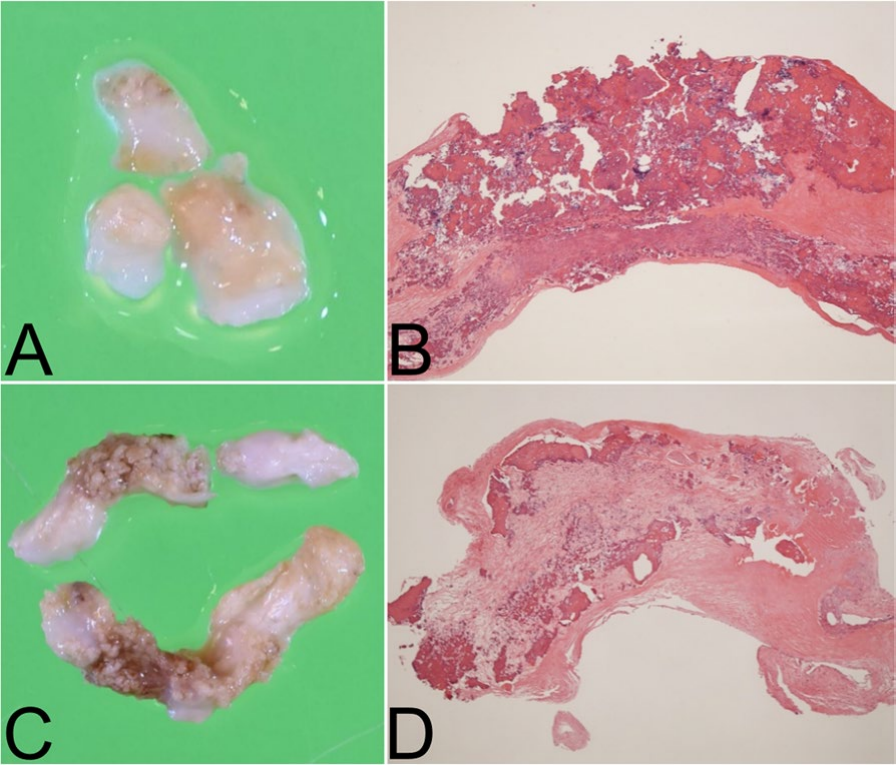
Gross and histopathological appearances of the resected masses. Gross appearance **A**, **C** and pathologic examination **B**, **D** of resected masses in the left atrium **A**, **B** and left ventricle outflow tract **C**, **D**; magnification: **B**, **D** × 20

Figure 3 shows macroscopic and histological examinations of the LA (Fig. 3A and B) and LVOT (Fig. 3C and D) masses. Macroscopic examination revealed that the LA mass was a calcified nodule (Fig. 3A), whereas the LVOT mass was club-shaped, yellowish-white, and partially calcified (Fig. 3C). Histological examination indicated that the CATs had different characteristics. The LA mass had nodular calcifications in dense fibrous tissue (Fig. 3B). In contrast, the LVOT mass included organized nodular calcifications surrounded by amorphous material, consisting of fibrin and sanguineous deposits, mixed with inflammatory macrophage infiltrate (Fig. 3D).

The patient was discharged without heart failure symptoms, and postoperative echocardiography revealed no recurrence of the cardiac masses after 2 years.

The patient consented to her medical details being disclosed in this report. The study protocol was approved by Ome Municipal General Hospital ethics committee (approval number: 50).

## Discussion and conclusions

Cardiac CATs are infrequent, non-neoplastic cardiac tumors that were first described in 1997 [1]. Few cases of multiple CATs occurring simultaneously have been reported [4–6]. However, only one case of multiple CATs in different locations has been reported [6]. The origin and etiology of CATs remain poorly understood. A review of 44 cases reported that most CATs were found in patients with MAC (70%) or a history of hemodialysis (20%) [2]. Abnormal calcium metabolism, because of renal dysfunction and inflammation associated with hemodialysis, may be linked to their growth and pathology; some reports have implicated thrombogenesis and hypercoagulability [7]. We described a case of two CATs simultaneously occurring in the LVOT and LA. The patient underwent tumor resections and aortic valve replacement for aortic regurgitation due to large cusp perforations.

Macroscopical and histological examinations showed that the tumors had distinct characteristics. Characteristics of both CATs have been reported in the previous literature. CATs, in most cases, showed similar pathological characteristics to the LVOT tumor in the present case, and a few tumors are similar to the LA tumor [4, 5, 8–11]. Particularly, it has been reported that all CATs occurring in LV and LVOT have similar characteristics to the LVOT tumor. We assumed these differences may suggest different stages of CAT development. CAT growth was reported as rapid, especially in patients with MAC, and associated with hemodynamic forces [3, 8]. Since the hemodynamic force in the left ventricle is stronger than that in the LA, this force in the left ventricle would cause early rupture of premature tumors and inhibit tumor growth, whereas tumors in the LA tend to grow larger and firmer because of weak hemodynamic forces. Some reports indicated that the hemodynamic force has an effect on vascular remodeling, plaque development, and vulnerability; however, no report evidenced the effect of hemodynamic force on CAT development [12, 13]. Matsukuma et al. reported three cases of CAT and indicated that the hemodynamic force could have an effect on CAT development [8].

Highly mobile CATs like the LVOT mass have been previously reported to have substantial risks of embolization [4, 8, 14]. Kinugasa et al. reported that a highly mobile CAT arose from MAC and caused perforation of the anterior leaflet and chordal rupture of the mitral valve resulting in mitral regurgitation [14]. No cases of other valve destruction have been reported previously. In our case, the LVOT mass swung widely and protruded through the aortic valve. Intraoperative findings indicated that perforations in coronary cusps caused aortic regurgitation that was absent in the previously conducted echocardiography, prior to CAT formation. Therefore, mechanical damages by the LVOT mass could have caused the cusp perforations that resulted in aortic regurgitation.

General therapy for CATs is tumor resection, though some cases can be treated conservatively [2]. CATs have a low risk of recurrence and carry good prognoses. It is difficult to distinguish CATs from other cardiac masses, such as thrombi, vegetation, myxoma, and osteosarcoma, based on preoperative examinations. Highly mobile CATs also have risks of embolization and mechanical injury to the surrounding tissues. Therefore, surgical resection is required to establish a more definitive diagnosis and prevent complications.

Herein, we reported a rare case of two CATs simultaneously occurring in the LA and LVOT. The tumors had different characteristics on macroscopic and histological examination, suggesting different developmental stages. Highly mobile CATs need early resection because of the high risks for embolization and injuries to the surrounding tissues.

## Data Availability

The datasets used during the current study are available from the corresponding author on reasonable request.

## Abbreviations

CAT: Calcified amorphous tumors
LA: Left atrium
LVOT: Left ventricular outflow tract
MAC: Mitral annular calcification
